# Feasibility of Volatile Biomarker-Based Detection of Pythium Leak in Postharvest Stored Potato Tubers Using Field Asymmetric Ion Mobility Spectrometry

**DOI:** 10.3390/s20247350

**Published:** 2020-12-21

**Authors:** Gajanan S. Kothawade, Sindhuja Sankaran, Austin A. Bates, Brenda K. Schroeder, Lav R. Khot

**Affiliations:** 1 Department of Biological Systems Engineering, Washington State University, Pullman, WA 99164, USA; gajanan.kothawade@wsu.edu (G.S.K.); lav.khot@wsu.edu (L.R.K.); 2 Center for Precision and Automated Agricultural Systems, Washington State University, Prosser, WA 99350, USA; 3 Department of Entomology, Plant Pathology and Nematology, University of Idaho, Moscow, ID 83844-2329, USA; austinbates@uidaho.edu (A.A.B.); bschroeder@uidaho.edu (B.K.S.)

**Keywords:** potato storage, postharvest losses, rot detection, volatile compounds, FAIMS

## Abstract

The study evaluates the suitability of a field asymmetric ion mobility spectrometry (FAIMS) system for early detection of the Pythium leak disease in potato tubers simulating bulk storage conditions. Tubers of Ranger Russet (RR) and Russet Burbank (RB) cultivars were inoculated with *Pythium ultimum*, the causal agent of Pythium leak (with negative control samples as well) and placed in glass jars. The headspace in sampling jars was scanned using the FAIMS system at regular intervals (in days up to 14 and 31 days for the tubers stored at 25 °C and 4 °C, respectively) to acquire ion mobility current profiles representing the volatile organic compounds (VOCs). Principal component analysis plots revealed that VOCs ion peak profiles specific to *Pythium ultimum* were detected for the cultivars as early as one day after inoculation (DAI) at room temperature storage condition, while delayed detection was observed for tubers stored at 4 °C (RR: 5th DAI and RB: 10th DAI), possibly due to a slower disease progression at a lower temperature. There was also some overlap between control and inoculated samples at a lower temperature, which could be because of the limited volatile release. Additionally, data suggested that the RB cultivar might be less susceptible to *Pythium ultimum* under reduced temperature storage conditions. Disease symptom-specific critical compensation voltage (CV) and dispersion field (DF) from FAIMS responses were in the ranges of −0.58 to −2.97 V and 30–84% for the tubers stored at room temperature, and −0.31 to −2.97 V and 28–90% for reduced temperature, respectively. The ion current intensities at −1.31 V CV and 74% DF showed distinctive temporal progression associated with healthy control and infected tuber samples.

## 1. Introduction

Potato (*Solanum tuberosum* L.) is a nutritious food crop consumed worldwide for carbohydrates, amino acids, and essential vitamins [1]. However, tubers are highly susceptible to soilborne, bacterial, and fungal diseases that can cause considerable postharvest and economic losses [2,3,4]. Pythium leak or watery rot caused by the soilborne oomycetes, *Pythium ultimum* and *Pythium debaryanum* [5,6] can cause significant losses in storage. Pythium infections occur via wounds that result from harvest. Pythium leak readily develops as a result of high moisture in non-optimal storage conditions [6,7]. Pythium leak leads to crop losses during storage, transit, and sales [2]. In storage, effective control of this oomycete pathogen requires rapid and accurate tools for diagnosis, and earlier diagnostics may result in better management [8,9]. Visual inspection has been a common method to identify Pythium leak disease symptoms of grayish or brownish lesions with watery appearance around wounds [4]. The infected tuber tissue is cream-colored and turns brown when exposed to air. Such disease symptoms may not be visualized until the infection has progressed significantly and often too late to implement disease mitigation measures. In addition, there could be discrepancies and subjectivity involved in human inspections. Furthermore, visual approaches may be destructive and render limited sampling accuracies. Finally, there have been limited studies on non-destructive identification of the potato Pythium leak disease to aid in postharvest management [3].

The traditional methods used to identify, and discriminate Pythium species involve the microscopic inspection of morphological characteristics [10,11]. Taxonomic keys and descriptions have been developed and widely used for identifying Pythium species [12]. A loop-mediated isothermal amplification (LAMP) method has been developed for the rapid and accurate detection of *P. ultimum* in wheat, soybean, cucumber, and tobacco plants [11]. Proper design of primers is a significant constraint in LAMP assays [13]. Polymerase chain reaction (PCR) has been used as a tool to detect and quantify the causal agents of late blight (*Phytophthora infestans*), pink rot (*Phytophthora erythroseptica*), leak (*Pythium ultimum*), dry rot (*Fusarium sambucinum*), and soft rot (*Pectobacterium carotovorum* subsp. *Carotovorum*/*Pectobacterium atrosepticum*) in potato tubers [14,15]. Primers and probes have been designed for conventional and real-time quantitative PCR assays to detect all possible fungal and oomycete pathogens causing pink rot, watery wound rot, and gangrene in potatoes [16].

Gas chromatography–mass spectrometry (GC-MS) has been used for volatile organic compounds (VOCs) profiling to differentiate potato tubers infected with the dry and soft rot pathogens [17]. Stored potatoes infected with storage rot pathogens produce VOCs that could be proportional to the disease severity of physical damage [18,19]. The specificity of VOCs released after infection reflects upon its metabolism and the set of defenses activated by the host [20]. Such VOCs have been monitored through gas analysis of the storage environment [19,20,21,22,23]. The electronic nose technology has also been used for the early detection of soft rot disease in potatoes [24,25].

Specific to VOC profiling, field asymmetric ion mobility spectrometry (FAIMS) has been used for the identification of soft rot progression in potato tubers [19,21]. FAIMS works on the principle of a gas-phase separation technique that separates chemical-related ions based on their mobility [26]. A typical FAIMS system consists of two parallel electrodes and a detector plate located beyond the electrodes. The VOCs headspace with carrier gas is streamed between the electrodes in the direction of the detector plate, resulting in separation of the VOCs analyte constituents (ions) as they are subjected to two perpendicular electric fields pertinent to electrodes [27]. These electric field intensities range between 1 to 20 kV cm^−1^ [28]. The varying electric field pushes ions toward one of the electrodes and prevents them from reaching the detector plate, while a compensating voltage applied to either electrode enables ions of different mobility patterns to reach the detector plate [27,29]. Overall, the ion current mobility is a function of compensation voltage [30,31]. Unlike conventional GC-MS systems, FAIMS has the capability to operate under atmospheric pressure with relatively low sample preparation and analysis time requirements [32]. In addition, FAIMS has the potential for automated measurement of the VOCs associated with biological samples [33]. VOCs’ diagnostics offers a non-invasive, real-time, and automated tool to detect the diseases at the asymptomatic stage [34,35,36] before incurring storage losses. Previous studies reported the applicability of FAIMS technology in the detection of different storage diseases, such as soft rot in potatoes and sour skin rot of onion [19,21,22,23]. In addition, such technology has been used for low power mobile chemical separation and detection [37] and discrimination and quantification of isomeric trisaccharides in honey [38]. Furthermore, studies are required to check the applicability of FAIMS to automatically analyze, quantify, and characterize the VOCs specific to the storage infections for real-time applications. An automated VOC analysis system can be developed if the sensor systems, such as FAIMS, can be adapted to identify the potential biomarkers indicative of early infection in a storage facility and integrated with their air circulation system.

Non-destructive tools for detecting Pythium leak rot are currently unavailable, although numerous potato storage diseases have been detected using the gas analysis techniques. Additionally, the impact of Pythium leak is not well understood for numerous russet-skinned potato cultivars grown in the Pacific Northwest region of the United States [39]. Therefore, this study aims to detect the Pythium leak in stored Ranger Russet (RR) and Russet Burbank (RB) potatoes using FAIMS. Specific objectives are to: (1) evaluate FAIMS applicability for early detection of Pythium leak caused by *P. ultimum* at room temperature (25 °C) and reduced temperature conditions (4 °C); and (2) monitor the temporal progression of disease symptoms through FAIMS response characterizations.

## 2. Materials and Methods

### 2.1. Potato Tubers

Tubers of Ranger Russet (RR) and Russet Burbank (RB) representing cultivars with high production acreage in Washington State (USA) were obtained from cooperating growers in the Columbia Basin of Washington after harvest in the 2019 season. They were first visually inspected for any cuts or greening due to an increase in the glycoalkaloid compound solanine [40]. Healthy tubers were first washed with tap water and then with a sodium hypochlorite solution (diluted at 1:10 ratio with water) and allowed to air dry. Potatoes were inoculated with *P. ultimum* mycelial plugs, and non-inoculated potato dextrose agar (PDA) plugs for healthy controls as described below.

### 2.2. Inoculum Preparation and Inoculation

**Figure 1:**
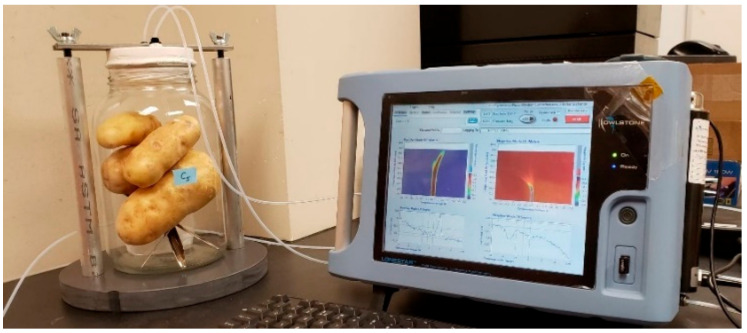
Experimental set-up used for volatile sampling data collection using the field asymmetric ion mobility spectrometry system.

The *P. ultimum* isolate (Pu-17) was grown on a PDA media (bacteriological grade agar at 15 g/L, potato dextrose broth at 24 g/L, Difco™ Casamino acids vitamin assay at 4 g/L) at ambient laboratory conditions with a 12 h On/Off UV light cycle. Pythium species have fine, colorless non-septate mycelia, reaching a diameter of 7 µm and form chlamydospores or oospores as overwintering structures [41]. A sterilized core borer of 7 mm size was used to produce plugs of media and mycelium. A sterile scalpel was then used to cut an approximate 2 cm plug out of the tuber. The mycelial plug was placed in the tuber wound, and the tuber material was returned to its original position. The scalpel was sterilized with cotton swabs soaked in 70% ethanol between each replicate consisting of 5 tubers. A separate scalpel was used for each replicate. A similar procedure was followed for control samples that were inoculated with a sterile PDA plug. Tubers were stored in a 3.78 L glass jar (Specialty Bottle, Seattle, WA, USA), which was top-sealed with a food-grade cling film to ensure aerobic storage conditions. A plastic petri dish (size: 95 × 15 mm, Fisher Scientific Company, LLC, Waltham, MA, USA) filled with sterile water was placed in the jars below the tubers (held in place by using a spatula set) to maintain the humidity (Figure 1). Glass jars containing potatoes were stored at room temperature (25 °C) or reduced temperature (4 °C). Tubers were periodically sprayed with sterile water to maintain high humidity in the jar to support disease development.

### 2.3. Experimental Design

Experiments involved four treatments (two temperature storage conditions: 4 °C and 25 °C, and two cultivars: RR and RB), each having two inoculation types, *P. ultimum* and PDA plug (as control). The reduced temperature (4 °C) was selected from a range of recommended cold storage temperatures (3–10 °C) [19,41]. Each treatment was replicated five times, and each replicate sample consisted of five tubers (weight: ~1 kg) that were inoculated with either *P. ultimum* PDA plug or non-inoculated PDA plug. Table 1 has pertinent experimental details. The experiments were divided based on storage temperatures; experiment-1 (RR) and experiment-2 (RB) at room temperature (25 °C), and experiment-3 (RR) and experiment-4 (RB) at reduced temperature (4 °C) conditions. The room temperature (25 °C) samples were stored in the laboratory. The reduced temperature (4 °C) samples were stored in a growth chamber. All the storage locations were sterilized with 70% ethanol solution before sample placement.

### 2.4. FAIMS Evaluations

**Figure 2:**
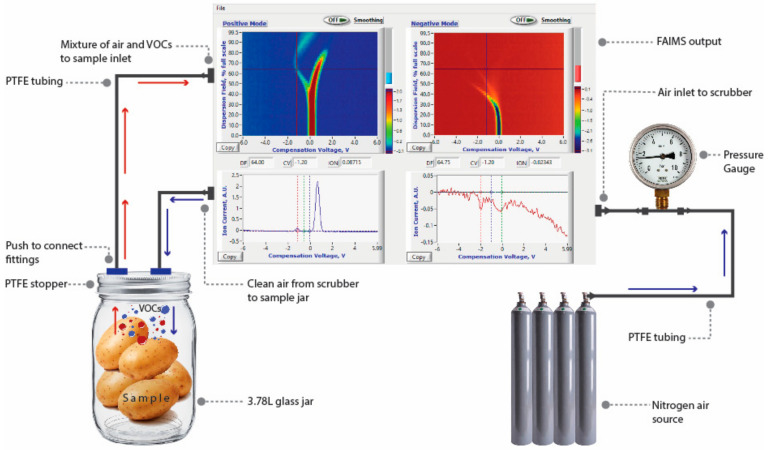
Schematic for the experimental set-up flow cycle used for volatile organic compounds analysis using portable field asymmetric ion mobility spectrometry system.

A portable FAIMS (Lonestar, Owlstone Nanotech Ltd., Cambridge, UK, Figure 1) system and a customized sampling unit were used for the headspace analysis in this study (Figure 2). The FAIMS working principle and operational details have been summarized previously [21]. Each sample glass jar was scanned to acquire three-dimensional data of 51 dispersion field (DF) intensities (0 to 100%), 512 compensation voltages (CVs) (−6 to 6 V), and ion currents (arbitrary units, AU). A customized headspace set-up (Figure 2) was designed to scan VOCs of sealed tight glass jar through a stopper. The stopper had two holes and was connected to the FAIMS ionization chamber using the polytetrafluoroethylene tubes (Figure 1). One tube carries nitrogen from the source to the jar (blue arrows) at a flow rate of 1.5 L min^−1^ and 60 kPa to flush out VOCs through the second tube (red arrows) to the analyzer (Figure 2). Each replicate was scanned four times using the FAIMS system and purged for about 30 to 40 min using nitrogen gas before the next sample scan.

The jars incubated at 4 °C were allowed to equilibrate to room temperature for an hour before FAIMS scanning. The sampling sequence of each replicate was randomized to avoid statistical biases. A blank sample jar (without tubers) was scanned through the system as a reference for each sampling day. FAIMS evaluations were conducted for each experiment at six-time points (Table 1). This amounted to a total of 264 scans per experiment (4 scans/replicate × 11 replicates (5 per treatment × 2 treatments + 1 blank) × 6 sampling days) and about 1056 scans in the entire study.

### 2.5. Data Analysis

**Figure 3:**
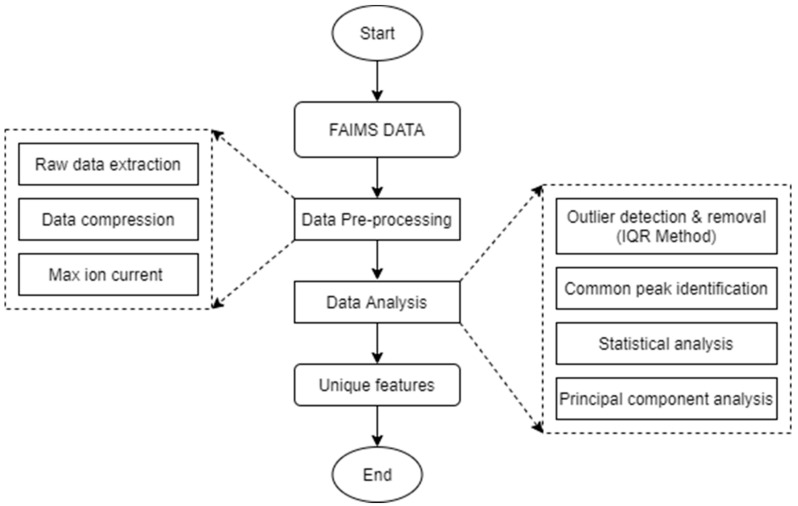
Field asymmetric ion mobility spectrometry data analysis flowchart. IQR refers to the interquartile range.

The general data analysis pipeline is summarized in Figure 3. Three-dimensional FAIMS data (CV, DF, and ion current) of each sample were first extracted (Figure 3) as the “*txt” files using the system’s software (Lonester, Owlstone Nanotech Ltd., Cambridge, UK). This data was transformed into 2-dimensions (all CV-DF combination and pertinent ion current values) for each scan using a custom-developed Python script (version 3.6). The transformed data was a matrix of 26,112 ion peaks (512 CV × 51 DF combination) as rows, and 44 samples ((5 replicate samples × 2 treatments + 1 blank sample) × 4 scans/replicate samples) as columns. The transformed concatenated matrix had ion current values (positive mode) of 26,112 ion peaks (for each CV-DF combination) for each sample as a data feature. The first scan of all replicate samples was excluded from further analysis. The ion current peaks—theoretically indicative of the released VOC ions—were then extracted from the transformed data using a moving CV window of 0.25 V that resulted in a matrix of 2448 CV-DF combination based on maximum ion current value for the specific CV window for each of 44 sample scans (Figure 3). Preliminary visualizations showed that peak ion currents differed between both the treatments (healthy control and *P. ultimum* inoculated), within the CV and DF ranges of −0.58 to −2.97 V and 30 to 90%, respectively for tubers stored at room temperature, and −0.31 to −2.97 V and 28 to 90%, respectively for the tubers stored at reduced temperature storage, respectively. Maximum ion currents pertinent to these ranges were extracted, thus creating a final matrix with ion current data of 28 CV-DF combinations in the above-mentioned range for about 30 sample scans ((5 replicate samples × 2 treatments) × 3 scans/replicate samples), resulting in a total of about 840 ion current data points. The interquartile range (IQR) method was then used to identify and replace the outliers with ‘Not-a-Number [NaN]’ values within the pre-processed data.

The common and unique CV-DF combinations were identified, and pertinent mean differences between the healthy control and *P. ultimum* inoculated tubers were analyzed using a two-sample *t*-test (α = 0.05). The CV-DF ion combinations that were present in both the healthy control and *P. ultimum* inoculated tuber samples were considered common CV-DF combinations. Meanwhile, the unique combinations were the ones that were present only in either *P. ultimum* inoculated or healthy treatment. Patterns of such differences between *P. ultimum* inoculated and healthy control samples were also recognized with principal component analysis (PCA) plots, and temporal disease progression was monitored using the Box–whisker plots (RStudio, Inc., Boston, MA, USA). For principal component analysis, the final data matric comprising of about 840 ion current data points (28 CV-DF combination × 30 sample scans) was utilized. The PCA plots were developed to observe the treatment (control and inoculated) differences in PC scores.

## 3. Results and Discussion

### 3.1. Salient FAIMS Signatures

#### 3.1.1. Room Temperature Storage Experiments

**Figure 4:**
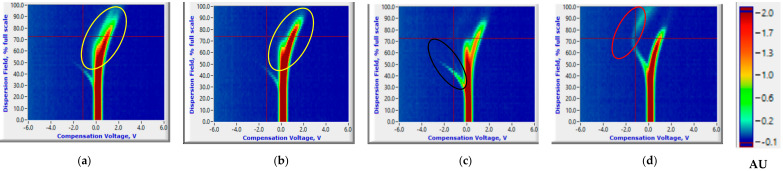
Ion current (AU) plots (corresponds to the color scale) at 0th (healthy control (**a**) and *P. ultimum* inoculated (**b**)) and 14th (healthy control (**c**) and *P. ultimum* inoculated (**d**)) days after inoculation for the Ranger Russet tuber sample replicates stored at room temperature (25 °C).

The FAIMS data with specific ion current ranges could consistently distinguish between healthy control and *P. ultimum* inoculated tuber treatments. Pertinent to this study, ion current peaks common to both healthy control and *P. ultimum* inoculated tubers were observed throughout the 14-day storage period (highlighted with a yellow ellipse, Figure 4a,b). The peaks curving to the left (highlighted with a black ellipse) are possibly the reactant ion peaks (RIPs) resulting from the nitrogen gas (Figure 4d). These peaks were distinguishably observed for the healthy controls and started disappearing for the *P. ultimum* inoculated samples as a result of Pythium leak related VOCs released in the later storage period. An increase in hydration can cause gradual changes in reactant ion peak profiles, and the flow of the gas/air, and the temperature can also cause changes in ion peak profiles [42]. The intensity of these RIPs could vary between the samples based on their biological composition or tissues modified during inoculation. The ion current peaks that were common for both healthy controls and *P. ultimum* inoculated treatments had significantly different mean values (Two-sample *t*-test, *p* < 0.05). However, this difference was not consistent and could not identify unique trends for either healthy control or *P. ultimum* inoculated samples (Supplementary Materials, Table S1).

**Figure 5:**
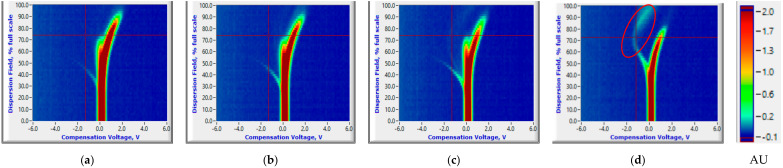
Ion current (AU) plots (corresponds to the color scale) at 0th (healthy control (**a**) and *P. ultimum* inoculated (**b**)) and 14th (healthy control (**c**) and *P. ultimum* inoculated (**d**)) days after inoculation for the Russet Burbank tuber sample replicates stored at room temperature (25 °C).

Ion current plots were identical for both healthy control and *P. ultimum* inoculated samples (Figure 4a,b or Figure 5a,b) on 0th day after inoculation (DAI). The peaks curving to the left and then top are entirely unique (highlighted with a red ellipse) and could be attributed to Pythium leak related VOCs carried by the nitrogen gas and were observed for both cultivars (Figure 4d or Figure 5d). Similar peaks were observed for potato soft rot associated VOCs by Rutolo et al. [22] and Sinha et al. [19,21,23]. Such peaks were not visible at 0th and 1st DAI and appeared later in the storage period (Figure 4d or Figure 5d). Previous studies [19,21] have also reported a similar disease detection time frame (1–3rd DAI) for samples stored at room temperature. Critical CV and DF intensity ranges of −0.57 to −2.97 V and 32 to 76%, respectively, showed the presence of the Pythium leak related VOCs (unique ion current peaks). For all DAIs, the ion currents attributing to the unique peaks were significantly lower for infected samples of RR and higher for RB (Two-sample *t*-test, *p* < 0.05) than healthy controls (Supplementary Materials, Table S2). Moreover, the number of such unique ion current peaks for RR was significantly larger than RB. This phenomenon might be attributable to the varying number of VOCs released by each of the two cultivars. The number of Pythium leak-related peaks also increased with the DAI, indicating the temporal progression of disease in infected samples (Supplementary Materials, Tables S3 and S4).

#### 3.1.2. Reduced Temperature Storage Experiments

**Figure 6:**
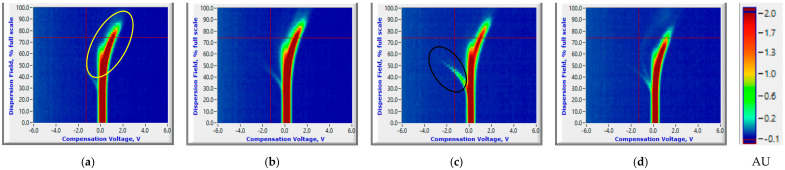
Ion current (AU) plots (corresponds to the color scale) at 0th (healthy control (**a**) and *P. ultimum* inoculated (**b**)) and 15th (healthy control (**c**) and *P. ultimum* inoculated (**d**)) days after inoculation for the Ranger Russet tuber sample replicates stored at reduced temperature (4 °C).

**Figure 7:**
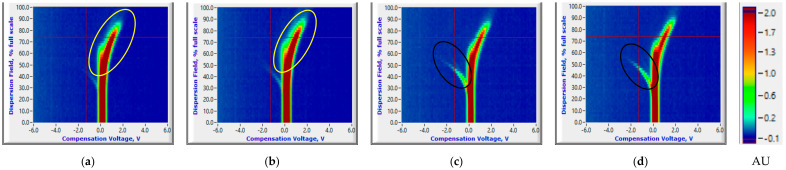
Ion current (AU) plots (corresponds to the color scale) at 0th (healthy control (**a**) and *P. ultimum* inoculated (**b**)) and 15th (healthy control (**c**) and *P. ultimum* inoculated (**d**)) days after inoculation for the Russet Burbank tuber sample replicates stored at reduced temperature (4 °C).

VOC signatures for healthy control and *P. ultimum* inoculated samples were nearly the same on 0th until 10th DAI (Figure 6a,b or Figure 7a,b), similar to the room temperature storage condition. The common ion current peaks for the healthy controls and *P. ultimum* inoculated tuber samples were observed throughout the 31-day storage period. The RIPs from carrier gas were also clearly observed for the healthy controls and disappeared for the infected samples at a lower rate than those under room temperature (Figure 6 and Figure 7). Some RIPs were also observed in *P. ultimum* inoculated RR samples later in the storage period, potentially due to utilization of reactant ions in carrying the VOCs pertinent to Pythium leak (highlighted with a red ellipse, Figure 7d). However, no such change in RIPs was observed for the RB replicates, where some RIPs were observed until late in the storage period (15th DAI). Although the spectral differences between *P. ultimum* inoculated and healthy control samples were not clearly visible, mean ion currents of common peaks were significantly different (Two-sample *t*-test, *p* < 0.05, Supplementary Materials, Table S5) for RR. The differences were not significant for RB and could be attributed to the cultivar response at reduced temperatures and possibly a slow or unsuccessful inoculation (Supplementary Materials, Table S6). Salas et al. [43] and Hollingshead et al. [36] assessed the incidence and severity of *P. ultimum* in potato cultivars and observed less severity and incidence of the leak in RB compared to RR. An increasing trend was observed for both unique and common ion peaks for RR cultivars, while very few peaks were observed for RB cultivar (Supplementary Materials, Tables S7 and S8). In general, *Pythium* infections are severe at high temperatures [40], and reduced storage conditions would have slowed the rate of infection and disease development. Previous studies also reported that storage temperature and time significantly affect the lesion expansion of the leak [17,36].

### 3.2. Pattern Recognition

#### 3.2.1. Room Temperature Storage Experiments

**Figure 8:**
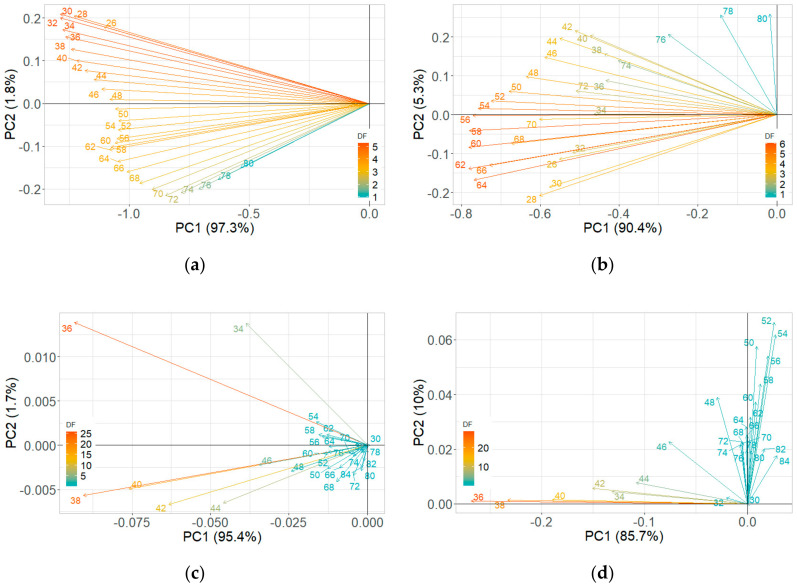
Principal component analysis loading plots for ion current of Ranger Russet tuber samples stored at room temperature (25 °C, experiment-1) on (**a**) 0th and (**b**) 14th days after inoculation, and Russet Burbank tuber samples stored at room temperature (25 °C, experiment-2) on (**c**) 0th and (**d**) 14th days after inoculation.

**Figure 9:**
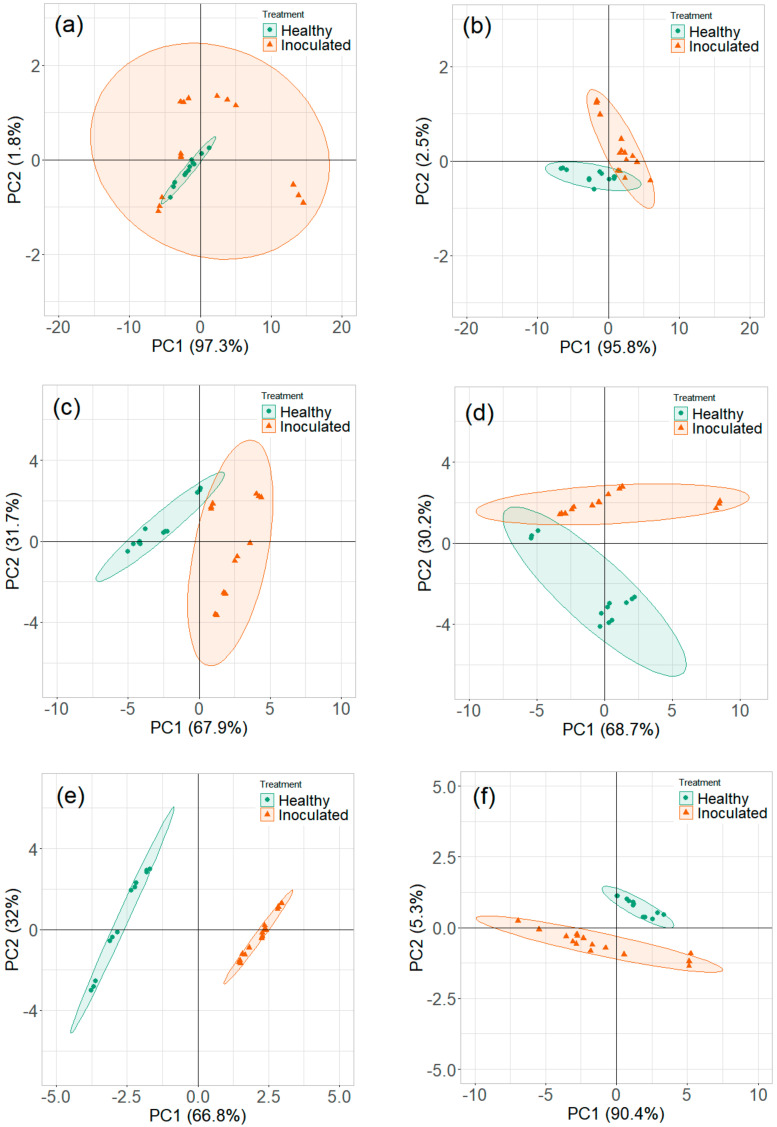
Principal component analysis plots for ion current of Ranger Russet tuber samples stored at room temperature (25 °C, experiment-1) on (**a**) 0th, (**b**) 1st, (**c**) 3rd, (**d**) 5th, (**e**) 7th, and (**f**) 14th days after inoculation.

**Figure 10:**
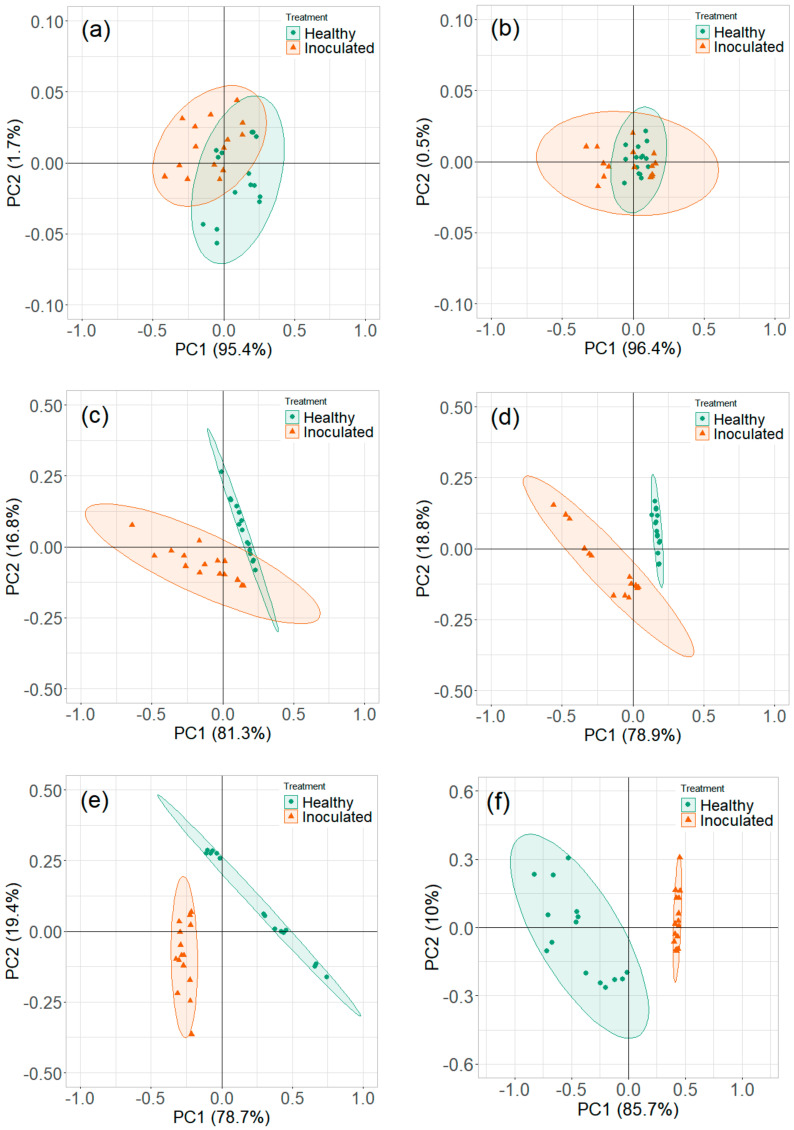
Principal component analysis plots for ion current of Russet Burbank tuber samples stored at room temperature (25 °C, experiment-2) on (**a**) 0th, (**b**) 1st, (**c**) 3rd, (**d**) 5th, (**e**) 7th, and (**f**) 14th days after inoculation.

The PCA plots of maximum ion current were extracted at each DF clusters of ion currents. The contribution of the DF intensities in the critical CV range is depicted in the PCA loading plot. The DF intensities differed in having more weight contributed towards the first two PCs (Figure 8). This DF range confirms the critical range observed in the ion current plots. The first two PCs accounted for nearly 97% of the variability within ion current (at CV-DF combination) datasets related to the healthy control and infected tuber samples (Figure 9 and Figure 10). Similar variability was reported by Rutolo et al. [22] in a study of potato soft rot detection. Cluster patterns were not separated on 0th DAI (Figure 9a) but were distinct after 1st DAI (Figure 9b); this explains consistent ion current differences between healthy control and infected tuber samples. Separated clusters were observed from 3rd to 14th DAI for the RR cultivar (Figure 9c–f) and the RB cultivar (Figure 10c–f). The clusters pertinent to healthy control samples were more compact than infected samples, most probably due to very low sample variance. Compact data points within the clusters belong to multiple scans of a sample replicate, indicating a similar VOC release pattern. It may also be inferred that different replicates within a treatment may release a different number of VOCs, possibly due to different bacterial colony growth in tubers [16,19,44].

#### 3.2.2. Reduced Temperature Storage Experiments

**Figure 11:**
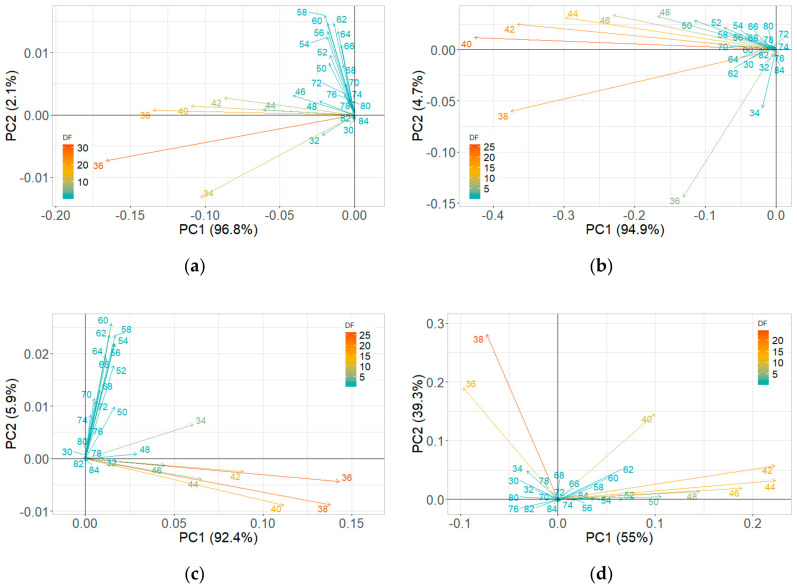
Principal component analysis loading plots for ion current of Ranger Russet tuber samples stored at reduced temperature (4 °C, experiment-3) on (**a**) 0th and (**b**) 31st days after inoculation and Russet Burbank tuber samples stored at reduced temperature (4 °C, experiment-4) on (**c**) 0th and (**d**) 31st days after inoculation.

**Figure 12:**
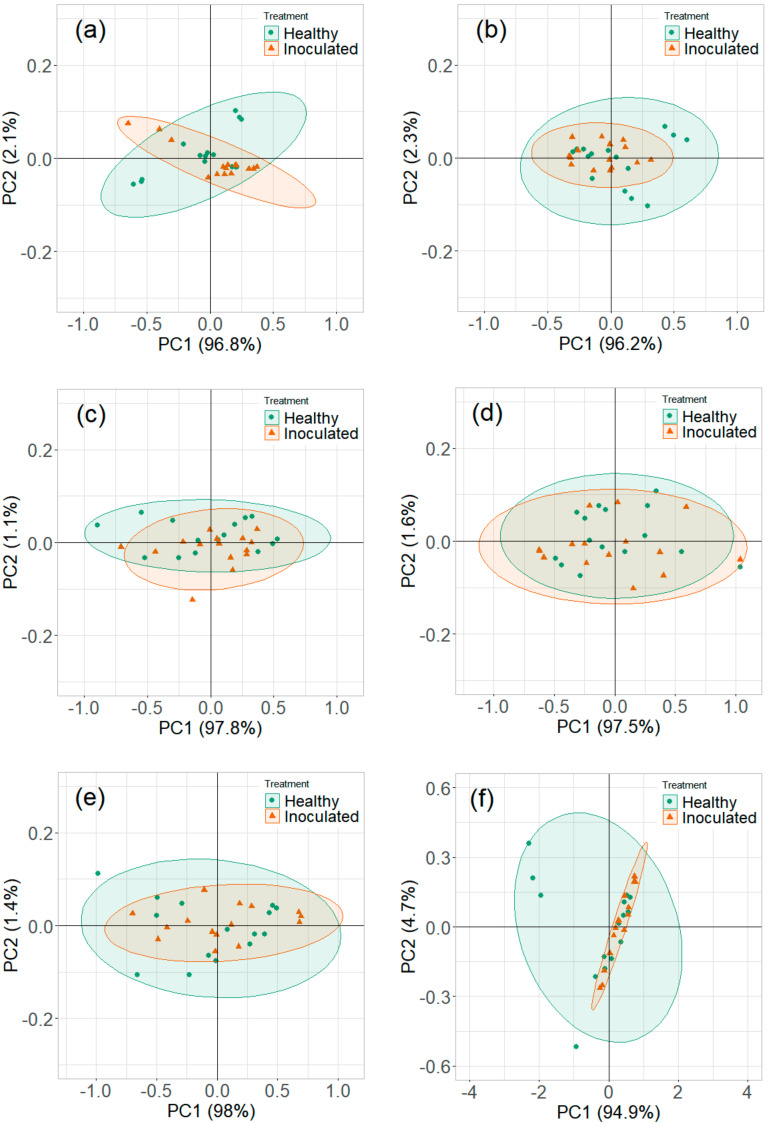
Principal component analysis plots for ion current of Ranger Russet tuber samples stored at reduced temperature (4 °C, experiment-3) on (**a**) 0th, (**b**) 1st, (**c**) 5th, (**d**) 10th, (**e**) 15th, and (**f**) 31st days after inoculation.

**Figure 13:**
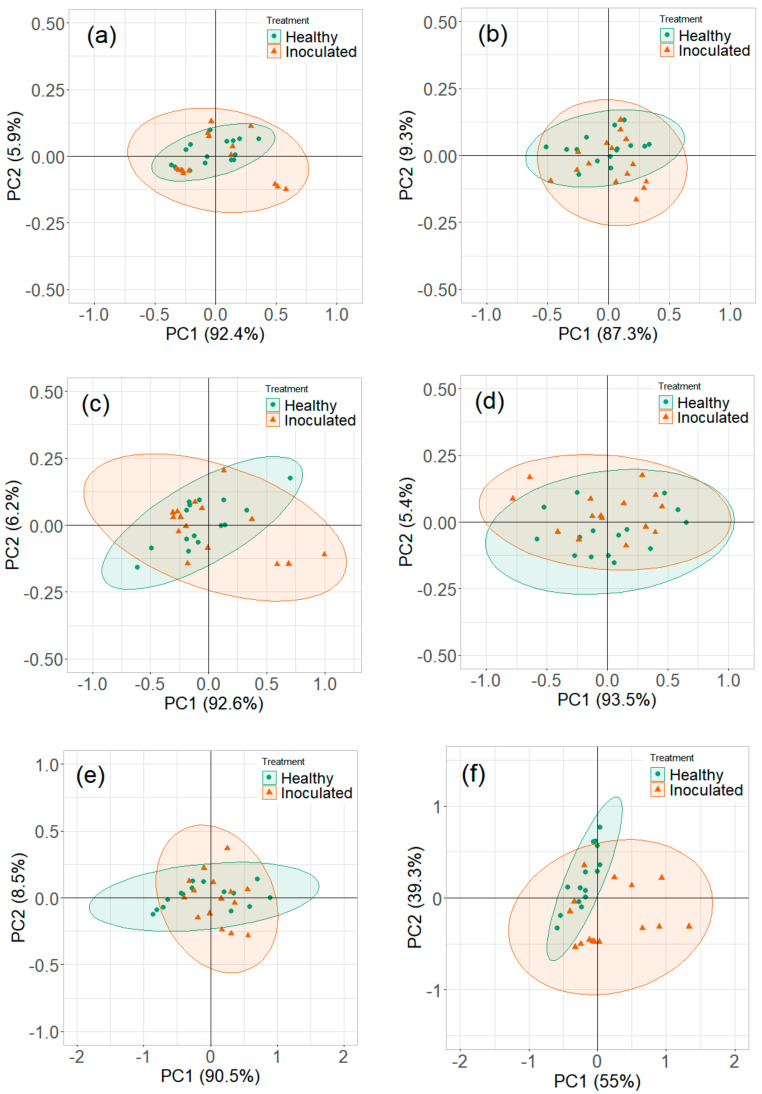
Principal component analysis plots for ion current of Russet Burbank tuber samples stored at reduced temperature (4 °C, experiment-4) on (**a**) 0th, (**b**) 1st, (**c**) 5th, (**d**) 10th, (**e**) 15th, and (**f**) 31st days after inoculation.

The contribution of variables was observed in the loading plots of PCs and variables (Figure 11). The contributing variables did not show a consistent pattern, although these variables were from the identified detection range. The PCA plots did not show a clear distinction between the healthy controls and *P. ultimum* inoculated tuber samples stored at reduced temperature (Figure 12 and Figure 13). Some data points that appeared far from the respective cluster could be attributed to the biological and storage conditions that may have different effects on different potato tubers [22]. The unclear distinction between healthy and *P. ultimum* inoculated samples also indicates a minimal progression of the Pythium leak under reduced temperatures while a very high growth under high temperatures (>18 °C) typical to harvest and postharvest (https://www.potatogrower.com/). Similar observations were reported for brown rot disease detection in potatoes under low storage temperatures [45]. Overall, VOC release appears to be affected by the storage temperature and having control over it may reduce postharvest crop losses. Studies have also reported the recovery potential of infected potato tubers when cooled below 18 °C (https://www.potatogrower.com/). El-Marzoky [46] also observed the lowest growth of *P. ultimum* isolates on PDA at 5 °C. The leak’s overall infection reduction could be the effect of the exposure of the pathogen to lower air temperature around the tuber shortly after the inoculation [17,36]. Storage temperatures below 18.3 °C are also reported as advantageous to limit bruise and remove field heat in typical storage designs to avoid Pythium leak infection [37]. Similarly, Lui et al. [17] observed no disease developed for the tubers stored at 4 to 8 °C for ≤45 days.

### 3.3. Temporal Progression

#### 3.3.1. Room Temperature Storage Experiments

**Figure 14:**
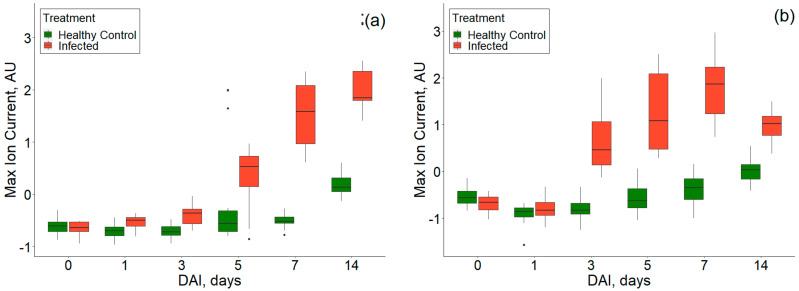
Ion current at 74% of the dispersion field and compensation voltage of −1.31 Volts showing the temporal progression of healthy and *P. ultimum* inoculated treatments for (**a**) experiment-1 and (**b**) experiment-2.

The progression of VOCs for healthy control and *P. ultimum* inoculated tuber samples was observed on a temporal scale of 0–14 DAI at CV, and DF intensity of −1.31 V and 74%, and consistent differences were also observed in ion currents (Figure 14). Overall, the ion currents for infected tuber samples were higher than healthy controls, and differences were highest on 7th DAI for both cultivars. This was also in accordance with the standard disease detection time frame (5th–7th DAI, [22]). After 7th DAI, the ion current continued to increase for RR but decreased for RB. Additionally, the ion current for infected tubers showed high variability with DAI than healthy controls [21]. Overall, the VOC release increases with the DAI.

#### 3.3.2. Reduced Temperature Storage Experiments

**Figure 15:**
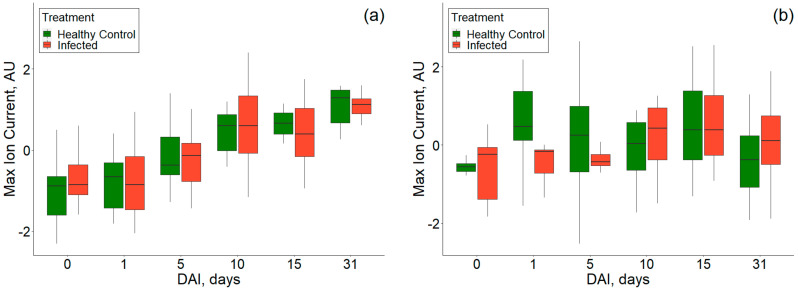
Ion current at 74% of the dispersion field and compensation voltage of −1.31 Volts showing the temporal progression of healthy and *P. ultimum* inoculated treatments for (**a**) experiment-3 and (**b**) experiment-4.

The Box–whisker plot shows the comparison of healthy control and *P. ultimum* inoculated tubers on a temporal scale of 0–31 DAI (Figure 15). On 0th DAI, the ion currents for *P. ultimum* inoculated RR samples were lesser than the healthy control samples while they were higher for the *P. ultimum* inoculated RB samples. However, the differences were negligible for reduced disease susceptibility at reduced temperature storages also observed by Biondi et al. [45] for potato brown rot. The mean ion currents were also observed to increase later in the storage period (Figure 15a). Unlike room temperature stored samples, non-uniform trends in VOC release were observed for RB (Figure 15b), indicating its lower susceptibility at such temperatures. Previous studies also reported that the disease severity is generally low at temperature <10 °C [43,47,48]. The extent of disease development is often determined by the pathogenicity and virulence of the primary pathogen present in the host. Lui et al. [17] observed reduced mycelial growth and low lesion expansion potential at a temperature of less than 12 °C. Reduced pathogenicity indicates the virulence or aggressiveness may be very nominal under reduced temperatures [49]. Olson et al. [50] reported that the pathogenicity of the Pythium species in infected snap beans from pod to seedling stage differed. Recently, Adnan et al. [51] studied the effect of temperature on the colony growth of *Pythium* spp. on zucchini and observed that the mycelial growth was limited at 10 °C.

## 4. Conclusions

This study evaluated field asymmetric ion mobility spectrometry towards early detection of the Pythium leak disease in stored potatoes at two storage temperatures, room temperature (25 °C) and reduced temperature (4 °C), respectively. FAIMS was able to detect VOCs resulting from Pythium leak (*P. ultimum*) inoculated potato tubers at an asymptomatic stage, i.e., as early as 1st DAI for both cultivar tubers stored at the room temperature (25 °C). For reduced temperature storage (4 °C), FAIMS detected disease symptom-specific VOC-biomarkers at around 5th DAI. The delayed detection is most likely due to the limited mycelial growth of the pathogen at lower temperatures. The FAIMS-based ion current data demonstrated pertinent changes in VOC concentration at critical CV and DF intensity ranges of −0.57 to −2.97 V and 32–76% for room temperature −0.57 to −2.97 V and 34–84% for reduced temperature storage conditions, respectively. The PCA at this critical CV-DF response combination ranges successfully aided in monitoring the disease progression. The PCA loading analysis confirmed the contributing range towards discrimination in the dataset. The greatest pathogenic activity was observed in the tubers stored at room temperature. Limited infection potential at low temperature [17,36,51] resulted in limited VOC release, where the differences in FAIMS spectral patterns between control and inoculated samples were not prominent. Temporal progression of the VOCs released from the healthy control and inoculated tuber samples was captured by the Box–Whisker plots at −131 V CV and 74% DF intensity, which showed consistent differences in both the classes.

Overall, the FAIMS response could suitably detect and quantify VOC biomarkers associated with Pythium leak and the temporal progression in stored potatoes, especially at room temperature. Nevertheless, knowing the leak susceptibility of the cultivars at bulk storage conditions and associated VOC biomarkers could help to develop potential cultivar-specific solutions for early detection and control of such disease under bulk storage conditions towards minimized postharvest losses.

## Figures and Tables

**Table 1:** Experimental details of conditions used for data collection using field asymmetric ion mobility spectrometry system.

Exp.	Cultivar	Inoculation	Replicates	Storage Conditions		Sampling Days (DAI)
				Temperature (°C)	Humidity (%)	
1	RR	*P. ultimum*	5	25	30	0, 1, 3, 5, 7, 14
	RR	sterile PDA plug	5	25	30	
2	RB	*P. ultimum*	5	25	30	
	RB	sterile PDA plug	5	25	30	
3	RR	*P. ultimum*	5	4	95	0, 1, 5, 10, 15, 31
	RR	sterile PDA plug	5	4	95	
4	RB	*P. ultimum*	5	4	95	
	RB	sterile PDA plug	5	4	95	

Exp: Experiment number, DAI: Days after inoculation, RR: Ranger Russet, RB: Russert Burbank, *P. ultimum*: *Pythium ultimum*, PDA: Potato Dextrose Agar.

## Supplementary Materials

The following are available online at https://www.mdpi.com/1424-8220/20/24/7350/s1. Table S1. Ion current (Mean ± Std. Error, AU) pertaining to common peaks at critical dispersion field (DF) intensity (25 °C). DAI, CV, RR, and RB refer to days after inoculation, compensation voltage, Ranger Russet, and Russet Burbank, respectively. Table S2. Ion current (Mean ± Std. Error, AU) pertaining to unique peaks at critical dispersion field (DF) intensity and critical compensation voltage range (−0.57 to −2.97 V) (25 °C). DAI, RR, and RB refer to days after inoculation, Ranger Russet, and Russet Burbank, respectively. Table S3. The critical DF intensities in −0.57 to −2.97 V compensation voltage range (25 °C). DAI, RR, and RB refer to days after inoculation, Ranger Russet, and Russet Burbank, respectively. Table S4. Common and unique peaks identified between the treatments on the sampling days (25 °C). DAI, RR, and RB refer to days after inoculation, Ranger Russet, and Russet Burbank, respectively. Table S5. Ion current (Mean ± Std. Error, AU) pertaining to common peaks at critical dispersion field intensity (4 °C). DAI, RR, and RB refer to days after inoculation, Ranger Russet, and Russet Burbank, respectively. Table S6. Ion current (Mean ± Std. Error, AU) pertaining to unique peaks at critical dispersion field intensity and critical compensation voltage range (−0.57 to −2.97 V) (4 °C). DAI, RR, and RB refer to days after inoculation, Ranger Russet, and Russet Burbank, respectively. Table S7. Critical dispersion field intensities in −0.57 to −2.97 V compensation voltage range (4 °C). DAI, RR, and RB refer to days after inoculation, Ranger Russet, and Russet Burbank, respectively. Table S8. Common and unique peaks identified between the treatments on the sampling days (4 °C).
