# Supplementary material for: Feasibility of Volatile Biomarker-Based Detection of Pythium Leak in Postharvest Stored Potato Tubers Using Field Asymmetric Ion Mobility Spectrometry
Source: Sensors (Basel). 2020 Dec 21;20(24):7350. doi: 10.3390/s20247350 (PMC7767497; doi:10.3390/s20247350)
Supplement: Supplementary file 1 [file sensors-20-07350-s001.pdf]

sensors

Supplementary Materials

Article

Feasibility of Volatile Biomarker-Based Detection of Pythium Leak in Postharvest Stored Potato Tubers Using Field Asymmetric Ion Mobility Spectrometry

Gajanan S. Kothawade [1,2], Sindhuja Sankaran [1,2,*], Austin A. Bates [3], Brenda K. Schroeder [3] and Lav R. Khot [1,2,*]

1. Department of Biological Systems Engineering, Washington State University, Pullman, WA 99164, USA; gajanan.kothawade@wsu.edu (G.S.K.); lav.khot@wsu.edu (L.R.K.)
2. Center for Precision and Automated Agricultural Systems, Washington State University, Prosser, WA 99350, USA
3. Department of Entomology, Plant Pathology and Nematology, University of Idaho, Moscow, ID 83844-2329, USA; austinbates@uidaho.edu (A.B.); bschroeder@uidaho.edu (B.K.S.)

* Correspondence: sindhuja.sankaran@wsu.edu (S.S.)

Table S1. Ion current (Mean ± Std. Error, AU) pertaining to common peaks at critical dispersion field (DF) intensity (25 °C). DAI, CV, RR, and RB refer to days after inoculation, compensation voltage, Ranger Russet, and Russet Burbank, respectively.

DAI	Experiment−1 (RR)					Experiment−2 (RB)				
	**CV**	**DF**	**Healthy control**	***P. ultimum* inoculated**	***p* -value**	**CV**	**DF**	Healthy control	*P. ultimum* inoculated	*p* -value
0	1.06	74	2.57 ± 0.06	1.98 ± 0.22	0.02	0.89	74	2.74 ± 0.15	2.22 ± 0.07	< 0.001
1	1.06	74	0.31 ± 0.01	0.19 ± 0.01	< 0.001	1.06	74	2.44 ± 0.05	1.95 ± 0.11	< 0.001
3	1.06	74	2.68 ± 0.04	1.88 ± 0.04	< 0.001	0.82	74	2.91 ± 0.13	2.08 ± 0.15	< 0.001
5	1.06	74	1.87 ± 0.06	2.53 ± 0.15	< 0.001	1.06	72	1.66 ± 0.15	2.27 ± 0.06	< 0.001
7	1.06	74	2.13 ± 0.10	1.70 ± 0.02	< 0.001	1.32	74	0.26 ± 0.02	0.65 ± 0.02	< 0.001
14	0.85	74	1.15 ± 0.04	1.24 ± 0.14	0.58	0.78	74	0.83 ± 0.12	0.32 ± 0.07	< 0.001

Table S2. Ion current (Mean ± Std. Error, AU) pertaining to unique peaks at critical dispersion field (DF) intensity and critical compensation voltage range (−0.57 to −2.97 V) (25 °C). DAI, RR, and RB refer to days after inoculation, Ranger Russet, and Russet Burbank, respectively.

DAI	Experiment−1 (RR)				Experiment−2 (RB)			
	**DF**	**Healthy control**	***P. ultimum* inoculated**	***p* -value**	**DF**	**Healthy control**	***P. ultimum* inoculated**	***p* -value**
0	80	−0.04 ± 0.00	−0.03 ± 0.00	0.01	58	−0.01 ± 0.00	0.01 ± 0.00	0.002
1	36	0.05 ± 0.01	0.15 ± 0.02	< 0.001	52	−0.03 ± 0.00	−0.02 ± 0.00	0.03
3	50	−0.04 ± 0.00	−0.02 ± 0.00	< 0.001	74	−0.03 ± 0.00	0.01 ± 0.00	< 0.001
5	34	0.01 ± 0.01	−0.03 ± 0.00	< 0.001	74	−0.03 ± 0.00	0.02 ± 0.01	< 0.001
7	74	−0.03 ± 0.00	0.03 ± 0.00	< 0.001	74	−0.02 ± 0.00	0.03 ± 0.01	< 0.001
14	74	−0.01 ± 0.00	0.07 ± 0.01	< 0.001	82	0.01 ± 0.00	0.06 ± 0.00	< 0.001

Table S3. The critical DF intensities in −0.57 to −2.97 V compensation voltage range (25°C). DAI, RR, and RB refer to days after inoculation, Ranger Russet, and Russet Burbank, respectively.

DAI	Experiment−1 (RR)	Experiment−2 (RB)
0	50, 54, 56, 58, 62, 80	34 to 58, 62, 64, 70
1	34 to 52, 64	46,52
3	44 to 50	34,36,40 to 84
5	34, 36	34 to 38, 40 to 84
7	34, 36, 40 to 84	30, 42, 44

14	32 to 46, 50 to 84	32 to 46, 52 to 60, 82, 84

Table S4. Common and unique peaks identified between the treatments on the sampling days (25˚C). DAI, RR, and RB refer to days after inoculation, Ranger Russet, and Russet Burbank, respectively.

DAI	Experiment−1 (RR)		Experiment−2 (RB)	
	unique peaks*	significant common peaks**	unique peaks*	significant common peaks**
0	3	68	2	62
1	12	24	3	18
3	3	90	8	108
5	23	30	17	91
7	70	132	27	24
14	81	29	32	45

*Pythium leak related unique peaks, **Common peaks: CV-DF combinations which are present in both treatments (healthy control and *P. ultimum* inoculated)

Table S5. Ion current (Mean ± Std. Error, AU) pertaining to common peaks at critical dispersion field intensity (4 °C). DAI, RR, and RB refer to days after inoculation, Ranger Russet, and Russet Burbank, respectively.

DAI	Experiment−3 (RR)					Experiment−4 (RB)				
	CV	DF	Healthy control	*P. ultimum* inoculated	*p* -value	CV	DF	Healthy control	*P. ultimum* inoculated	*p* -value
0	0.78	74	0.14 ± 0.04	0.10 ± 0.02	0.31	0.78	74	0.99 ± 0.07	0.76 ± 0.06	0.02
1	0.78	74	0.14 ± 0.03	0.37 ± 0.05	< 0.001	1.06	74	1.7 ± 1.12	1.54 ± 0.09	0.34
5	0.78	74	−0.84 ± 0.07	−0.46 ± 0.07	< 0.001	1.06	74	1.6 ± 0.13	1.48 ± 0.08	0.44
10	0.78	74	−0.86 ± 0.10	−0.40 ± 0.11	< 0.001	1.06	74	1.53 ± 0.14	1.28 ± 0.1	0.18
15	0.78	74	−2.31 ± 0.06	−1.80 ± 0.11	< 0.001	1.32	74	0.57 ± 0.08	0.34 ± 0.04	0.02
31	1.32	74	−3.83 ± 0.15	−4.17 ± 0.08	0.06	1.32	74	1.59 ± 0.13	1.32 ± 0.12	0.14

Table S6. Ion current (Mean ± Std. Error, AU) pertaining to unique peaks at critical dispersion field intensity and critical compensation voltage range (−0.57 to −2.97 V) (4 °C). DAI, RR, and RB refer to days after inoculation, Ranger Russet, and Russet Burbank, respectively.

DAI	Experiment−3 (RR)				Experiment−4 (RB)			
	DF	Healthy Control	*P. ultimum* inoculated	*p* -value	DF	Healthy control	*P. ultimum* inoculated	*p* -value
0	-	-	-	-	-	-	-	-
1	34	0.08 ± 0.01	0.05 ± 0.01	0.02	32	−0.02 ± 0.00	−0.03 ± 0.00	0.032
5	54	−0.01 ± 0.00	−0.02 ± 0.00	0.003	-	-	-	-
10	66	−0.02 ± 0.00	−0.02 ± 0.00	0.02	34	0.18 ± 0.01	0.22 ± 0.01	0.049
15	62	−0.02 ± 0.00	−0.02 ± 0.00	0.007	78	−0.03 ± 0.00	−0.02 ± 0.01	0.043
31	44	0.04 ± 0.1	0.14 ± 0.02	0.04	38	0.8 ± 0.05	0.42 ± 0.06	< 0.001

Table S7. Critical dispersion field intensities in −0.57 to −2.97 V compensation voltage range (4˚C). DAI, RR, and RB refer to days after inoculation, Ranger Russet, and Russet Burbank, respectively.

DAI	Experiment−3 (RR)	Experiment−4 (RB)
	DF	DF
0	-	-
1	34, 60, 84	32, 74
5	52, 54, 58, 84	-
10	66	30,34,58,62,70,72
15	30, 56, 60, 62, 64, 68, 70	78
31	32, 38 to 46, 56, 58, 62, 66	34, 36, 38

Table S8. Common and unique peaks identified between the treatments on the sampling days (4˚C).

**DAI**	**Experiment−3 (RR)**		Experiment−4 (RB)	
	**unique peaks***	**significant common peaks****	**unique peaks***	**significant common peaks****
0	73	0	4	27
1	154	22	3	0
5	104	77	0	0
10	143	102	0	0
15	111	79	0	0
31	95	14	1	16

*Pythium leak related unique peaks, **Common peaks: CV-DF combinations which are present in both treatments (healthy control and *P. ultimum* inoculated)
